# Supplementary material for: Dopaminergic Signalling Enhances IL-2 Production and Strengthens Anti-Tumour Response Exerted by Cytotoxic T Lymphocytes in a Melanoma Mouse Model
Source: Cells. 2022 Nov 9;11(22):3536. doi: 10.3390/cells11223536 (PMC9688276; doi:10.3390/cells11223536)
Supplement: Supplementary file 1 [file cells-11-03536-s001.zip › cells-1887591-supplementary.pdf]

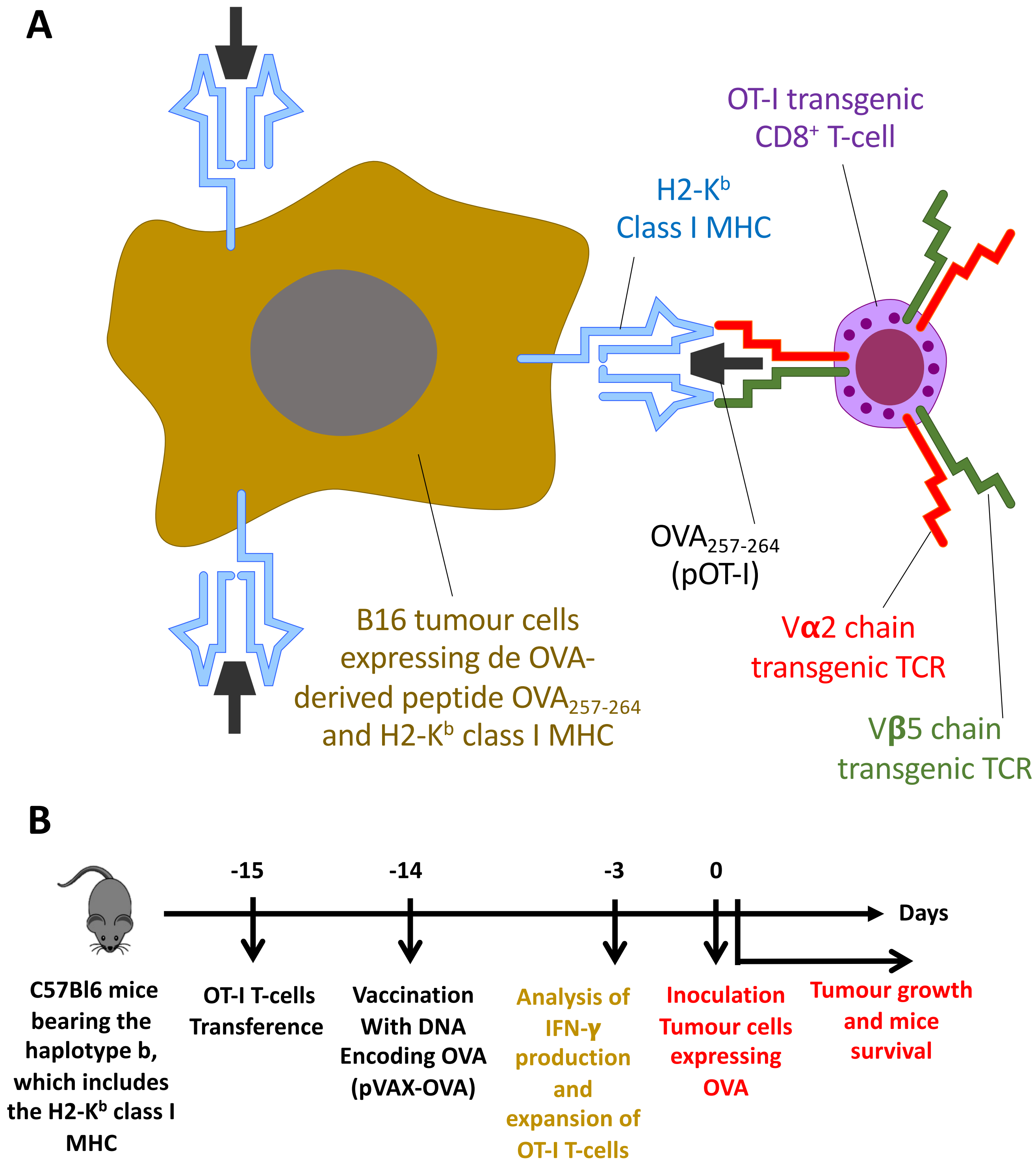

Figure S1. Schematic representation of the experimental system. (**A**) Illustration of how OT-I transgenic CD8$^+$ T-cells recognise the pOT-I over the H2-K^b molecules. The transgenic OT-I TCR might recognise the H2-K^b/pOT-I complexes expressed on the B16 tumour cells (depicted in the figure) or on the Surface of endogenous cells (not depicted) of C57Bl6 mice (which express H2-K^b molecules) upon the vaccination with OVA. (**B**) The timeline represents the experimental design of the results shown in figures 1 and 5. Black letters show common steps in both figures; Brown letters show the analysis performed in figure 1, whilst red letters represent the analysis conducted in figure 5.

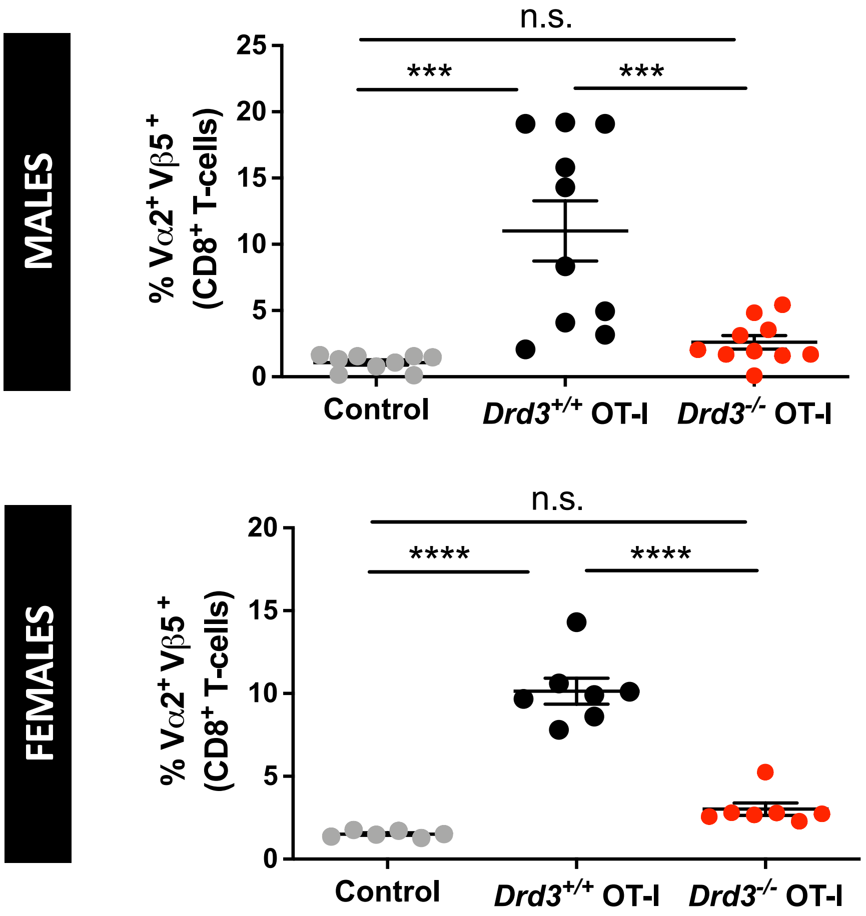

Figure S2. *Drd3*-deficiency results in attenuated $CD8^+$ T-cell expansion in both males and females. Splenic $CD8^+$ T-cells were isolated from *Drd3*$^{+/+}$ or *Drd3*$^{-/-}$ OT-I mice, and then i.v. transferred into C57BL/6 mice (2 x 10^5 cells/mouse). A group of mice did not receive the transfer of exogenous T-cells (Control). One day later, mice received an intradermal vaccination with 40µg of pVAX-OVA, and 11 days after vaccination, blood samples were obtained. The expansion of OT-I cells was evaluated separately in males (top panel) and females (bottom panel) as the percentage of cells expressing the transgenic TCR (Vα2 Vβ5) in the $CD3^+$ $CD8^+$ ZAq^- gate. Data from three independent experiments are shown. Data from 9-10 (males) or 6-7 (females) mice per group is shown. Each symbol represents data from an individual mouse. The mean ± SEM are depicted. *, p<0.05; **, p<0.01; ****, p<0.0001 by one-way ANOVA followed by Tukey's posthoc test. n.s., not significant.

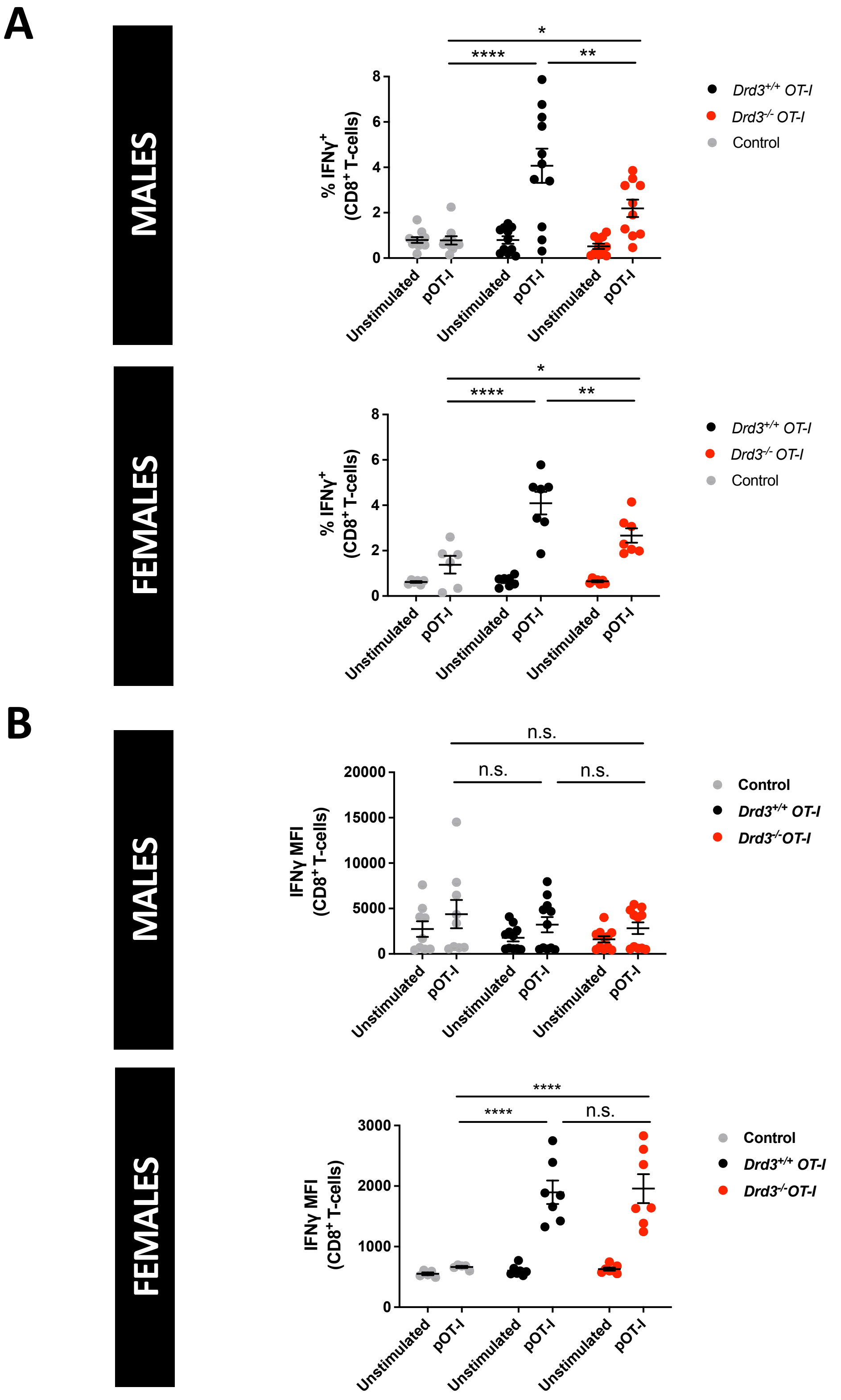

Figure S3. *Drd3*-deficiency results in a reduced percentage of CD8$^+$ T-cells producing IFN-γ in both males and females. Splenic CD8$^+$ T-cells were isolated from *Drd3$^{+/+}$* or *Drd3$^{-/-}$* OT-I mice, and then i.v. transferred into C57BL/6 mice (2 x 10^5 cells/mouse). A group of mice did not receive the transfer of exogenous T-cells (Control). One day later, mice received an intradermal vaccination with 40μg of pVAX-OVA, and 11 days after vaccination, blood samples were obtained. Cells were left unstimulated or stimulated with pOT-I (1 μg/mL) for 6 h, and the intracellular production of IFNγ was evaluated in the CD8$^+$ T-cell population by flow cytometry. Quantification of the percentage of IFNγ$^+$ cells (**A**) and the MFI associated with IFNγ$^+$ (**B**) in the CD8$^+$ ZAq$^-$ gate are shown separately for males and females. Data from three independent experiments are shown. Data from 9-10 (males) or 6-7 (females) mice per group is shown. Each symbol represents data from an individual mouse. The mean ± SEM are depicted. *, p<0.05; **, p<0.01; ****, p<0.0001 by two-way (B) ANOVA followed by Tukey's posthoc test. n.s., not significant.

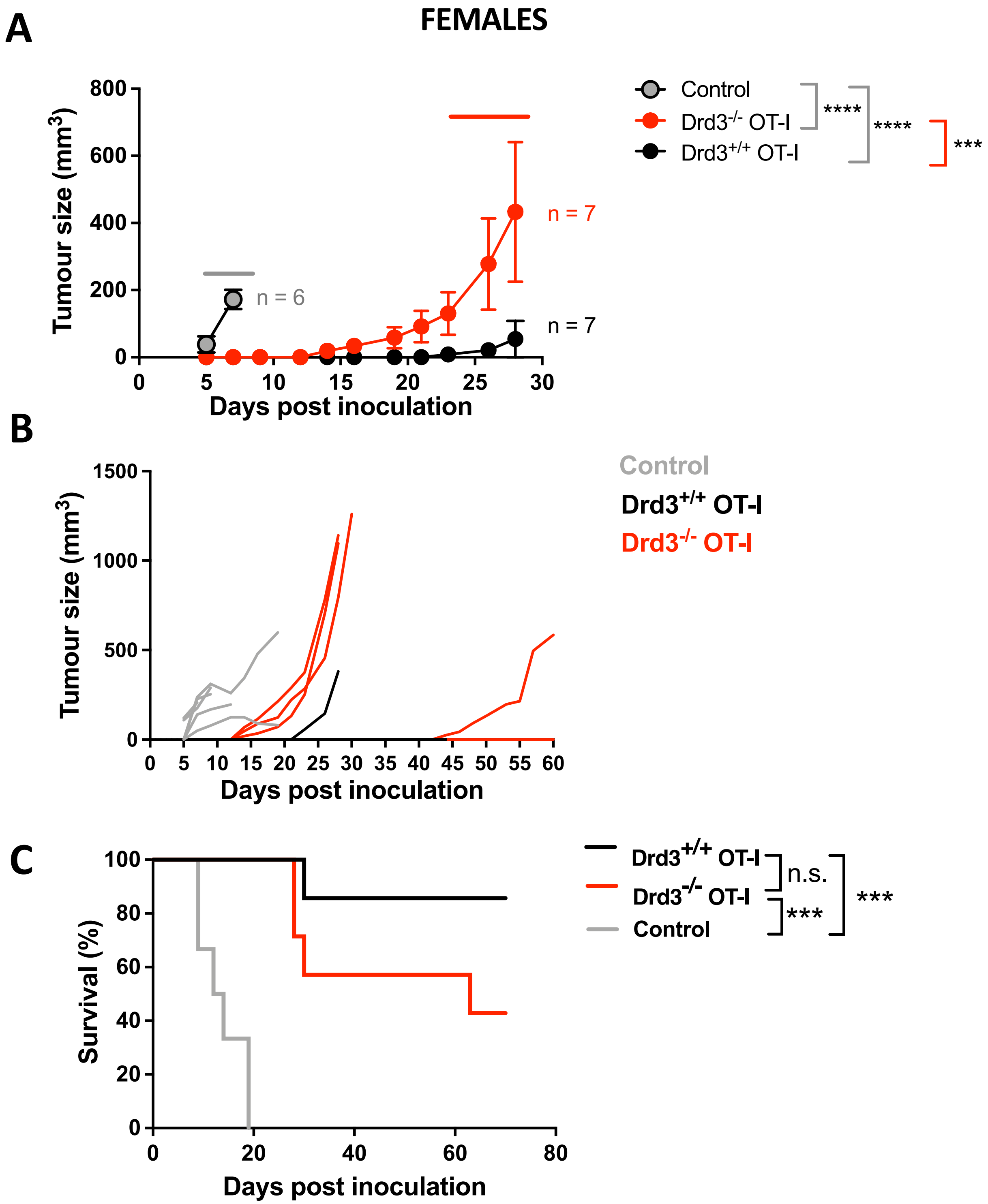

Figure S4. *Drd3*-deficient CD8⁺ T-cells exhibit reduced potency in the anti-tumour response in female mice. Splenic CD8⁺ T-cells were isolated from *Drd3*$^{+/+}$ or *Drd3*$^{-/-}$ OT-I mice, and then i.v. transferred into female C57BL/6 mice (2 x 10^5 cells/mouse). A group of mice did not receive the transfer of exogenous T-cells (Control). One day later, mice received an intradermal vaccination with 40 µg of pVAX-OVA. Thirteen days after vaccination, mice were s.c. inoculated with B16/OT-I melanoma cells (10^6 cells/mouse), and tumour growth and mice survival were monitored over time. (**A**) Tumour growth is represented as tumour volume in time. Data is the mean ± SEM from five mice per group. The top grey line indicates the frame of time where significant differences were found compared to the control group, and the red line indicates the frame of time where significant differences were found between mice receiving *Drd3*$^{+/+}$ or *Drd3*$^{-/-}$ OT-I cells. (**B**) Tumour growth curves for individual mice are shown. (**C**) Mice's survival over time is shown. (A-C) Data from a representative out of two independent experiments is shown (n = 6-7 mice per group). **, p<0.01; ***, p<0.001 by two-way ANOVA followed by Tukey's posthoc test (A, grey line), by multiple t-test (A, red line), or by long-rank Mantel-Cox test (C). n.s., not significant.
